# An automated pipeline for bouton, spine, and synapse detection of in vivo two-photon images

**DOI:** 10.1186/s13040-017-0161-5

**Published:** 2017-12-20

**Authors:** Qiwei Xie, Xi Chen, Hao Deng, Danqian Liu, Yingyu Sun, Xiaojuan Zhou, Yang Yang, Hua Han

**Affiliations:** 1 Research Base of Beijing Modern Manufacturing Development, No.100, Pingleyuan, Beijing, 100124 China; 2 Data Mining Lab, School of Management, Beijing University of Technology, No.100, Pingleyuan, Beijing, 100124 China; 3 Institute of Automation, Chinese Academy of Sciences, 95 Zhongguancun East Road, Beijing, 100190 China; 4 Faculty of Information Technology, Macau University of Science and Technology, Avenida Wai Long, Taipa, Macau, China; 5 Institute of Neuroscience, Chinese Academy of Sciences, 320 Yue Yang Road, Shanghai, 200031 China; 6 Beijing Normal University, No. 19, Waida Jie, Xinjie Kou, Beijing, 100875 China; 7 Center for Excellence in Brain Science and Intelligence Technology Shanghai Institutes for Biological Sciences, Chinese Academy of Sciences, 320 Yue Yang Road, Shanghai, 200031 China; 8 University of Chinese Academy of Sciences, School of future technology, No.19(A) Yuquan Road, Beijing, 100049 China

**Keywords:** in vivo two-photon imaging, Synapse, Bouton, Spine, Image enhancement

## Abstract

**Background:**

In the nervous system, the neurons communicate through synapses. The size, morphology, and connectivity of these synapses are significant in determining the functional properties of the neural network. Therefore, they have always been a major focus of neuroscience research. Two-photon laser scanning microscopy allows the visualization of synaptic structures in vivo, leading to many important findings. However, the identification and quantification of structural imaging data currently rely heavily on manual annotation, a method that is both time-consuming and prone to bias.

**Results:**

We present an automated approach for the identification of synaptic structures in two-photon images. Axon boutons and dendritic spines are structurally distinct. They can be detected automatically using this image processing method. Then, synapses can be identified by integrating information from adjacent axon boutons and dendritic spines. In this study, we first detected the axonal boutons and dendritic spines respectively, and then identified synapses based on these results. Experimental results were validated manually, and the effectiveness of our proposed method was demonstrated.

**Conclusions:**

This approach will helpful for neuroscientists to automatically analyze and quantify the formation, elimination and destabilization of the axonal boutons, dendritic spines and synapses.

## Introduction

Synapses were first discovered in the 1890s, when Sir Sherrington, through his pioneering work on motor reflexes, wrote that synapse is the way of neuronal communication in the nervous system [1]. There are two major types of synapses: chemical and electrical. In the mammalian central nervous system, the vast majority of the synapses are chemical. Chemical synapses, especially excitatory synapses, typically consist of presynaptic axon boutons and postsynaptic dendritic spines. The structural plasticity of boutons and spines underlies functional synaptic plasticity, widely accepted as the neural basis of learning and memory. Brain imaging can be used to characterize changes occurring in a brain during very different time-scales [2]. The advent of boutons and spines can be imaged in live animals over days or even months, allowing observation of structural changes in vivo, often in direct association with learning [3–11].

Manual validation is extremely time-consuming, and error prone. Meanwhile, different criteria may lead to different results. Therefore, manual methods are not suitable for the processing of large-scale data. The recent advances in biomedical imaging have allowed the initial development of computer-aided semiautomatic or automatic approaches to detect dendritic spines based on image analysis. In [12], Xie et al. proposed an algorithm for automatic neuron reconstruction. The algorithm can handle complex structures adaptively and optimize the localization of bifurcations. In [13], an automated scheme to perform segmentation in a variational framework was proposed to trace neurons from confocal microscopy images. The segmentation framework, referred to as “tubularity flow field (TuFF)”, performs directional regional growing guided by the direction of tubularity of the neurites. In [14], a robust automatic neuron segmentation and morphology generation algorithm was proposed. The algorithm-Tree2Tree uses a local medial tree generation strategy in combination with a global tree linking to build a maximum likelihood global tree. It is a reliable technique to compare various of neurons for tracing evaluation and neuron retrieval. Gonzalez et al. presented an approach to fully automated delineation of tree structures in noisy 2D images and 3D image stacks. It is able to eliminate noise while retaining the right tree structure [15]. Besides, in [16], Gonzalez et al. showed that using steerable filters to create rotationally invariant features that include higher-order derivatives, and training a classifier based on these features allows us handle such irregular structures. Rodriguez et al. developed an open-source software NeuronStudio to aid the neuroscientist in the task of reconstruction of neuronal structures from confocal and multi-photon images [17]. It is a self-contained software package that is free, easy to use. The focus of previous work mentioned above varies, with some focusing on neuronal tracking, segmentation and others on specific situations.

They inspired us to explore 3D tracking, segmentation and extraction of synapses both in 2D and 3D based on the detection results of our automatic detection method. Therefore, it is of interest to explore methods of automatic detection and quantification of synapses, dendrites and axons.

In addition to examining boutons and spines separately by two-photon microscopy, it is also possible to visualize synaptic connections with identified boutons and spines that are in close proximity. Although the resolution of light microscopy is larger than the size of the synaptic cleft, previous studies have showed that over 85% of putative synapses identified in deconvoluted confocal images were true synapses confirmed using electron microscopy [18]. Light microscopy can still provide useful information. Given that boutons and spines originated from different brain regions or of different cell types can be labeled using different fluorescent proteins, observation of synaptic connections using two-photon microscopy provides a valuable method for researching long-range and cell-type specific synaptic plasticity in vivo [19]. Therefore the automated detection of synapses will be of tremendous help for this kind of data analysis.

In this paper, we focus on the detection of axonal boutons, dendritic spines and synapses from the in vivo two-photon image stacks. As described above, a synapse typically consists of one axonal bouton and one dendrite spine, with the exception of multi-bouton and multi-spine synapses. A reasonable strategy to locate the synapses is to first detect axonal boutons and dendritic spines, then to search for synaptic contacts composed of bouton and spine pairs. A robust Gaussian model was used in order to enhance the morphology of axonal boutons and dendrites respectively, while effectively inhibiting noise. Before the enhancement operation, we performed deconvolution on axon images as a preprocessing method for noise reduction. And the regions with relatively higher values are regarded as axonal boutons with great possibility. For the detection of dendritic spines, we performed one-threshold segmentation to obtain the structure of the dendrites based on the enhanced images of dendrites, which followed by an efficient thinning algorithm. After we extracted the centerline of the dendrites, the dendritic spines were determined by finding the bifurcation points and endpoints.

## Material and method

**Fig. 1:**
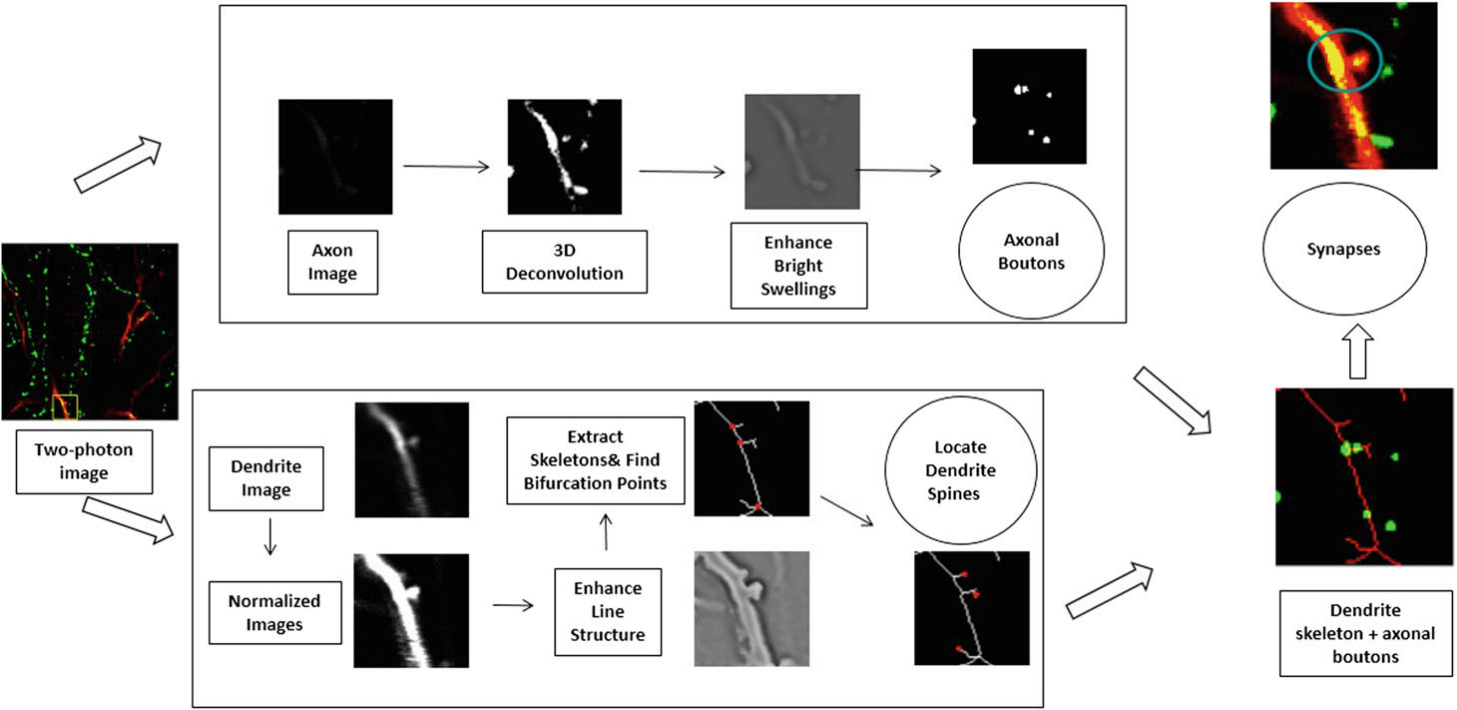
Workflow of detecting synapses on in vivo two-photon images of mouse

Figure 1 illustrates the workflow of our proposed approach for detection of synapses. We will give a detailed description of each procedure of the method after the introduction to the image stack in this paper.

### Materials

The image data used in this study was obtained from Institute of Neuroscience, State key Laboratory of Neuroscience, Chinese Academy of Sciences, Center for Excellence in Brain Science and Intelligence Technology. The transgenic mice (YFP-H line), both male and female, were imaged using a two-photon microscope (Sutter), controlled by Scanimage (Janelia). Auditory cortex of mice was exposed surgically and covered with glass cranial window for repeated two-photon imaging in vivo. Surgical details refer to Y. Yang [19]. Image stack was acquired from the cortical surface to 100–150 *μ*m depth with 0.7 *μ*m intervals. A 25 × objective with 1.05 numerical aperture was used (Olympus). A Ti:sapphire laser (Spectra-Physics) was used as the light source, and tuned to 92 nm for imaging. YFP (Yellow Fluorescent Protein) and GFP (Green Fluorescent Protein) signals were collected using filters 495/40 and 535/50 (Chrome). The 535/50 filter (Channel 1) collected both GFP and YFP signals, and the 495/40 filter (Channel 2) collected GFP-only signals. By subtracting the GFP signals from Channel 1 signals, the YFP-only images were obtained [19].

**Fig. 2:**
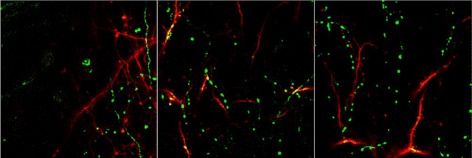
Two-photon images of mouse

The dual-color images, as shown in Fig. 2, are the two-photon images, where the red section and green section represent YFP (containing dendrites and axons) and GFP images (contains long-range projecting axons only) respectively and the spine-bouton pairs are thought to be synapse. The *x*-*y* resolution and the *z* resolution of the image data are 137 nm/pixel and 700 nm/pixel respectively, and the image size (*x*-*y*) is 512-by-512.

### Detection of axonal boutons

**Fig. 3:**
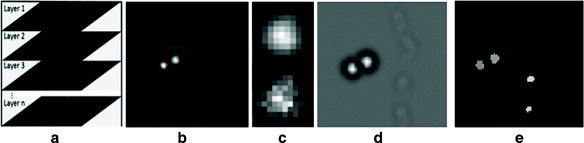
Work of detecting axonal boutons. **a** Axon image stacks from the two-photon image stacks. **b** One deconvolved axon image after 3D deconvolution. **c** Magnified bouton from the area indicated by the red rectangle shown in panel (**b**). The image is shown in two different states with the deconvolved one on the top and the original one on the bottom. **d** The enhanced image. **e** The final detected axonal boutons



In this section, we provide algorithmic details for axonal bouton detection. The proposed algorithm is divided into three parts. First, a 3D deconvolution operation is required due to the noise in the original image stacks. Next, we enhanced the bright swellings in the deconvolved images and segmented them. Finally, we identified true axonal boutons based on a series of criteria. The whole workflow for detecting axonal boutons is shown in Fig. 3.

#### 3D deconvolution

Although confocal microscopy images are known to be sharper than standard epifluorescence ones, they are still inevitably degraded by Poisson noise and residual out-of-focus light due to photon-limited detection [20]. Thus, several deconvolution methods have been proposed. In this study, we adopt the Deconvolution Approach for the Mapping of Acoustic Sources (DAMAS), which decouples the array design and processing influence from the noise being measured using a simple and robust algorithm [21]. The details of 3D deconvolution operation implemented in ImageJ [22] are shown in Appendix.

One deconvolved axon image is depicted in Fig. 3 b. To demonstrate the performance of the 3D deconvolution operation, we show an axonal bouton indicated by the red rectangle in Fig. 3 b. We then show two different states of this image in Fig. 3 c, with the deconvolved one on the top and the original one on the bottom. A significant difference can be seen from the detailed comparison, showing that the 3D deconvolution operation helps to identify the axonal boutons.

#### Enhancing bright swellings

The thresholding on a deconvolved image does not necessarily ensure perfect segmentations, or even good ones. This is because the range of the intensity of different axonal boutons can vary dramatically. A low threshold will reserve the bright axon shaft, but a high threshold will eliminate weak axonal boutons. To avoid the loss of data, we first enhanced the bright swellings.

**Fig. 4:**
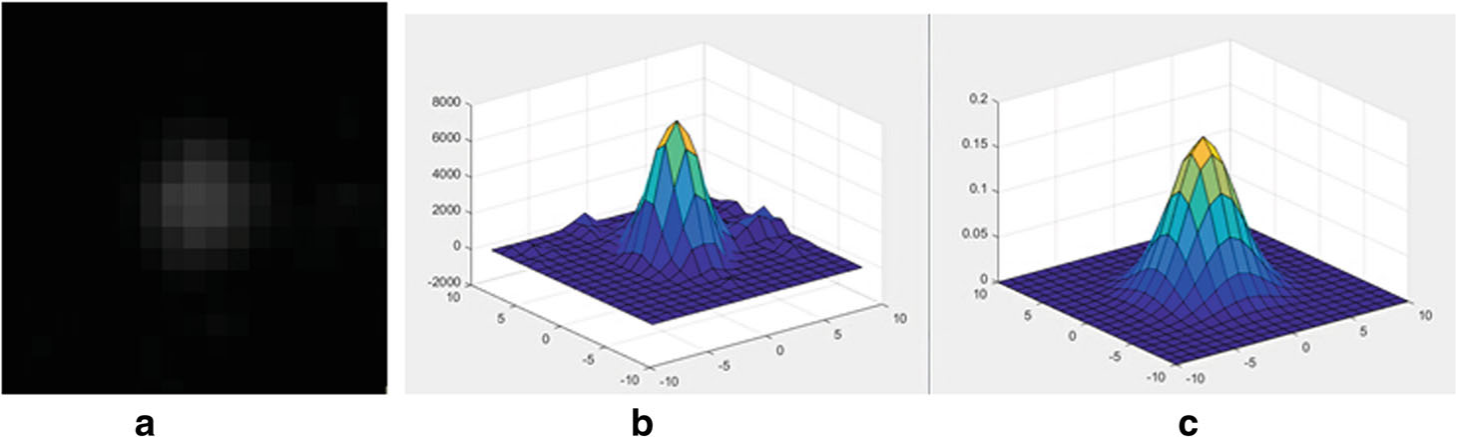
Axonal bouton intensity modeling. **a** A randomly selected axonal bouton. **b** The intensity image of the bouton shown in (**a**). **c** The three-dimensional Gaussian surface with a variance of 4/$\sqrt 3$

By the statistics carried on the corresponding electron microscope data, the average diameter of terminal boutons is 1.0 *μ*m. By setting the pixel size to 137 nm, we find the average radius of an axonal bouton is about 4 pixels. We randomly select an axonal bouton as shown in Fig. 4 a and show the plot of its corresponding intensity image in Fig. 4 b. Note that the axonal bouton is a “rounded” profile. We can see that the image in Fig. 4 b looks very similar to the three-dimensional Gaussian surface plotted in Fig. 4 c, suggesting it is reasonable to model the intensity of axonal bouton using a three-dimensional Gaussian surface,

1 $$ R(x,y) = C\exp\left(-\frac{{{{\left(x - {x_{0}}\right)}^{2}} + {{\left(y - {y_{0}}\right)}^{2}}}}{{2{\delta^{2}}}}\right),  $$

where *C* is a constant corresponding to the coordinate of the maximum magnitude point (*x*_0_,*y*_0_), and *δ* is the variance of the Gaussian surface. A very small part of the axonal boutons can be approximated by a ridge, we then construct a Hessian-based ridge detector. Let *m*=(*x*−*x*_0_)^2^+(*y*−*y*_0_)^2^. The intensity of enhanced image is set as additive inverse of the eigenvalue with minimum absolute, i.e. [Appendix A],

2 $$  \lambda (m) = \left\{{\begin{array}{c} { - \exp\left(- \frac{m}{{2{\delta^{2}}}}\right)\left(m - 2{\delta^{2}}+\sqrt {{{\left(m + 2{\delta^{2}}\right)}^{2}} - 4{\delta^{4}}} \right)/2{\delta^{5}},m \le 2{\delta^{2}}}\\ { - \exp\left(- \frac{m}{{2{\delta^{2}}}}\right)\left(m - 2{\delta^{2}} - \sqrt {{{\left(m + 2{\delta^{2}}\right)}^{2}} - 4{\delta^{4}}} \right)/2{\delta^{5}},m > 2{\delta^{2}}.} \end{array}} \right.  $$

Here we analyze in three cases:

- : *m*=0,*λ*(*m*)=1/*δ* ^3^;
- : $m = 2{\delta ^{2}},\lambda (m) = - \sqrt 3 /({\delta ^{3}} \times e),$ where *e* is the Euler’s number;
- : *m*→*∞*,*λ*(*m*)=0.

For the parabolic line profile, the magnitude of the second derivative of the extracted position is always maximum at the line position [23]. We can conclude that the relationship between the variance *δ* and the radius *r* of the axonal bouton is $\delta =r/\sqrt 3$ [Appendix B]. Combined with the radius of axonal boutons, we set the variance as $\delta =r/\sqrt 3$ in this study.

**Fig. 5:**
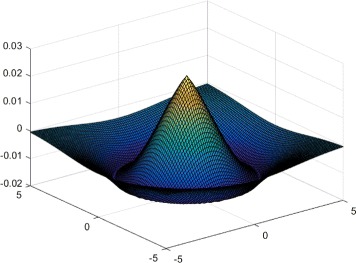
Eigenvalue of a matrix satisfying the distribution of model (1)

**Fig. 6:**
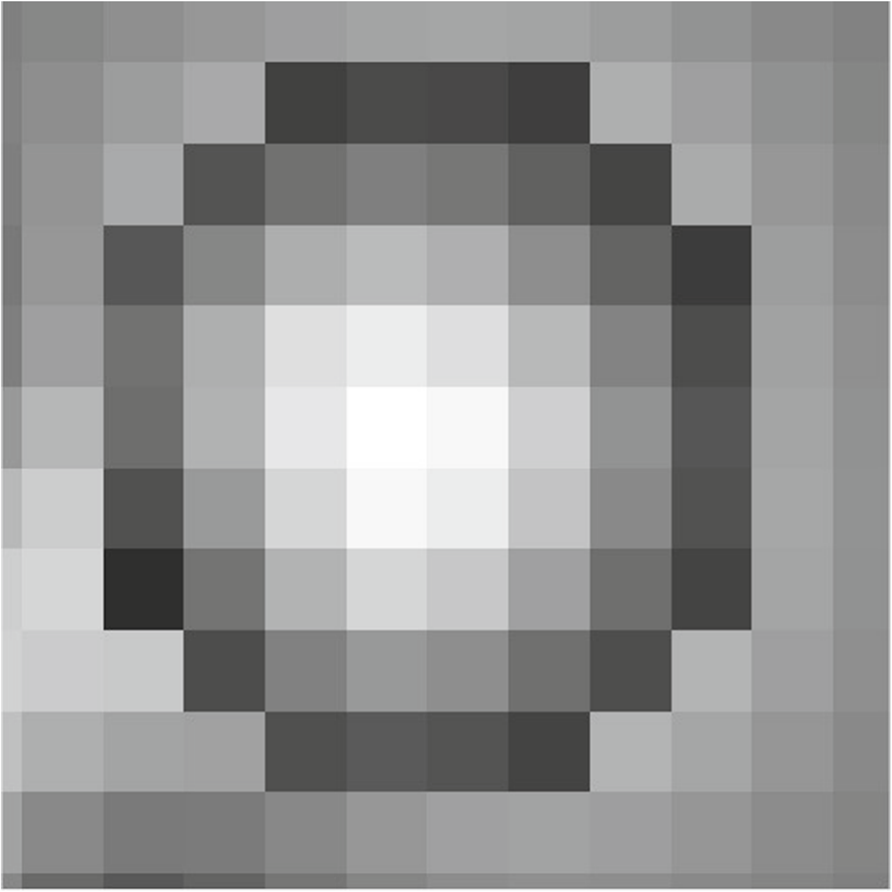
The enhanced bright swelling



To allow visual interpretation, we plot the chosen eigenvalue of model (1) in Fig. 5, from which we can see that the central region is enhanced while the surrounding region weakens gradually. This provides the theoretical basis for image enhancement and segmentation. Inspired by [23–25], we select the above variance. Figure 3 depicts the enhanced image of one bright swelling, whose variation tendency consistently conforms to that of Fig. 3 almost everywhere, supporting the correctness of our theoretical analysis. Compared to the image in Fig. 3 b, the enhanced image shown in Fig. 3 d has an advantage for weaker axonal boutons because of its more obvious profile. The following work is based on the enhanced image in Fig. 6, and the detail is stated in Algorithm 2.

#### Obtaining axonal boutons

As discussed in last section, the application of a relatively lower threshold will inevitably generate false positives. Fortunately, the shapes of the axonal boutons are homogeneous and each has a sole maximum point. Therefore, we first find local maximum points as candidate points for axonal boutons, a simple but effective strategy. The detail is stated in Algorithm 1.

We then evaluate whether each region in the resulting segmentation contains a local maximum points and we delete the regions lacking local maximum points. On this basis, we compute some statistical characteristics including the eccentricity, major axis, and minor axis. Then we reserve the regions that exhibit statistical characteristics similar to a disk. Finally, we record the location of the reserved regions and determine whether the peak intensity of each region is more than three times brighter than its axon shaft in the original image [19]. The final result of axonal boutons analysis is shown in Fig. 3 e.



### Detection of dendritic spines

**Fig. 7:**
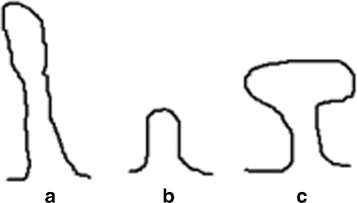
Common types of dendritic spines. **a** thin **b** stubby **c** mushroom

In this section, we explicate the details of our method for the detection of dendritic spines. Dendritic spines are small with spine head volumes ranging from 0.01 *μ* *m* ^3^ to 0.8 *μ* *m* ^3^. According to the shape, dendritic spines can be classified into following types: thin, mushroom, and stubby, as shown in Fig. 7 [26]. The variable shape of these spines is related to the strength and maturity of the synapses [27]. Thus, based on the forms, it is reasonable to locate the dendritic spines by looking for the spur pixels that are connected to the bifurcation points.

**Fig. 8:**
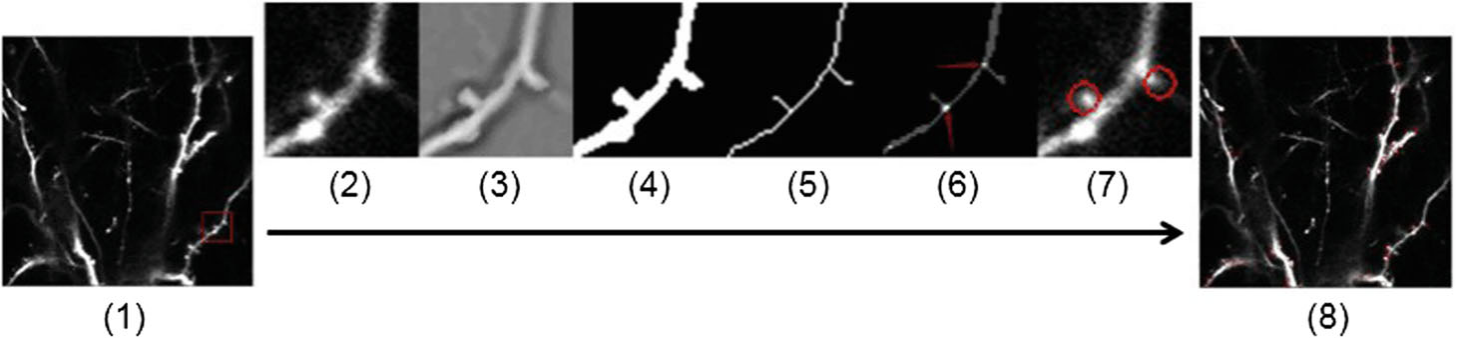
Workflow of detection of dendritic spines. (**1**) Normalized image of dendrite; (**2**) Region specified by red rectangle in (**1**); (**3**) Corresponding enhanced image; (**4**) Segmentation result; (**5**) Skeletons of dendrite; (**6**) Branch points on skeletons; (**7**) The finally detected result of dendritic spines; (**8**) Result of dendrite



The proposed algorithm consists of three parts: enhancement of the line structure in the images after pretreatment; segmentation of dendrites and extraction of their skeletons; and identification of the dendritic spines based on the dendritic skeletons. The workflow is shown in Fig. 8 and Algorithm 4.

#### Enhancing line structure

Before performing other operations, we first normalize the images to reduce the impact of noise by using the following formula:

3 $$ I(x,y) = \frac{I(x,y) - I_{min}}{I_{max} - I_{min}},  $$

where *I*(*x,y*) is the intensity value in *I* at (*x,y*), *I*_*max*_ and *I*_*min*_ represent the maximum intensity and minimum intensity value of the image respectively.

**Fig. 9:**
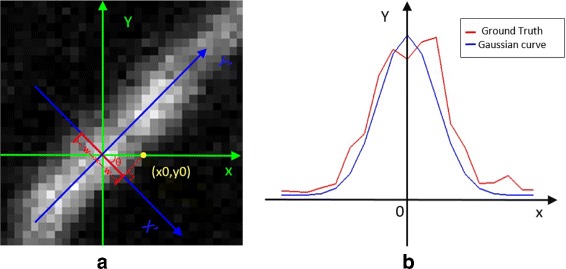
Dendrites intensity modeling. (**a**) Part of a dendrite, a section of which is marked with red. The *x*-*y* coordinates are marked with green and the Y axis in the Cartesian coordinate system marked with blue is the principle direction of the line structure; (**b**) Intensity value of the dendrite section marked by red line in (**a**) and the Gaussian curve with a variance of 2, marked with red and blue respectively

Next, we enhance the linear structure. As shown in Fig. 9, the intensity value of each section of the dendritic linear structure can be modeled as a Gaussian curve [23], which can be written as

4 $$ I(x') = {C_{den}}\exp\left(- \frac{{x'^{2}}}{{2{\sigma^{2}}}}\right) = {C_{den}}\exp\left(- \frac{{{{\left(x\cos \theta - y\sin \theta \right)}^{2}}}}{{2{\sigma^{2}}}}\right),  $$

where *x* ^′^ is the abscissa on Cartesian coordinate system *X* ^′^- *Y* ^′^; *x,y* are the abscissa and ordinate on Cartesian coordinate system *X*-*Y*, respectively; *C*_*den*_ is the maximum pixel value of the cross section; *σ* is the variance of the Gaussian curve, and *θ* is the angle between the cross section and the main direction of the linear structure, which is shown in Fig. 9 a. According to [23], we can obtain the relationship between the variance *σ* and the radius of the lines structure *w* [Appendix C]: $\sigma = w/\sqrt 3$.

The average diameter of the dendritic spine of the part is less than 0.9 *μ*m, while the *x*-*y* resolution is 137 nm/pixel, so the average radius *w* is equal to 3 pixels.

As in previous part, we construct a Hessian-based ridge detector and take the additive inverse of the eigenvalue with maximum absolute value as the intensity of the enhanced image [Appendix D]:

5 $$ I_{enh}(x,y) = \left\{ {\begin{array}{c} { - {\sigma^{2}}\lambda (x,y),~\text{if}~\lambda (x,y) < 0}\\ {0,~\text{otherwise}} \end{array}} \right.  $$

The approach for enhancing line structure can be summarized as follows:



#### Extracting skeleton and finding branch points

We use the following Algorithm 6 to get the dendritic skeleton *C* (Fig. 8 (5)) at the basis of the binary image *B* (Fig. 8 (4)), which is obtained by segmenting the enhanced image *I* (Fig. 8 (3)) using a suitable threshold [28]:

1. In the first sub-iteration, delete pixel *p* if and only if the condition (a), (b), (c) are all satisfied.
2. In the second sub-iteration, delete pixel *p* if and only if the condition (a), (b), (d) are all satisfied.

- Condition (a): *X*_*H*_(*p*)=1
  where ${X_{H}}(p) = \sum \limits _{i = 1}^{4}{b_{i}}$, ${b_{i}} = \left \{ {\begin {array}{*{20}{c}} { 1,~if~x_{2i-1} = 0~\text {and}~(x_{2i} = 1~or~x_{2i+1} = 1)}\\ { 0,~\text {otherwise}} \end {array}} \right.$ *x*_1_,*x*_2_,…,*x*_8_ are the values of the eight neighbors of *p*, starting from the east neighbor and numbered in counter-clockwise order.
- Condition (b): 2≤ min{*n*_1_(*p*),*n*_2_(*p*)}≤3,
  where ${n_{1}}(p) = \sum \limits _{k = 1}^{4} {{x_{2k - 1}} \cup {x_{2k}}}$, ${n_{2}}(p) = \sum \limits _{k = 1}^{4} {{x_{2k}} \cup {x_{2k + 1}}}$.
- Condition (c): $({x_{2}} \cup {x_{3}} \cup {\overline x_{8}}) \cap {x_{1}} = 0$
- Condition (d): $({x_{6}} \cup {x_{7}} \cup {\overline x_{4}}) \cap {x_{5}} = 0$

The two sub-iterations together make up one iteration of the algorithm and the iterations are repeated until the resulting image stops changing. The approach for extracting skeletons can be summarized as follows:



In this study, the operation for finding branch points is a two-dimensional convolution of the binary image of skeletons and a 3-by-3 filter, with an intensity value of 0 for 4 vertices and 1 for the rest positions. The points with an intensity value equal or greater than 4 are considered as branch points. Figure 8 (6) illustrates the branch point detection results.

#### Locating dendritic spines

**Fig. 10:**
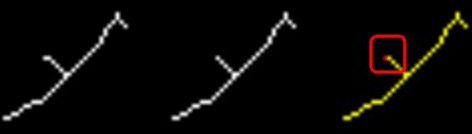
Illustration of locating dendritic spines. From left to right: Skeleton, skeleton after removing spur pixels, the overlapped image. The red point is the position of dendritic spine (within the red square) and the structure marked by yellow is the overlapped section of the skeleton and skeleton after removing spur pixels

We locate the suspected dendritic spines as follows: 1 〉 Remove spur pixels of the dendritic skeletons. The removed pixels are the putative locations of the dendritic spines. 2 〉 In a bifurcation-centered and properly sized region of skeleton, if there is an alternative point connected with the branch point, we consider the alternative point indicates the position of dendritic spine. This process is illustrated in Fig. 10 and the detail is stated in Algorithm 4.

#### Filtering points on axons

**Fig. 11:**
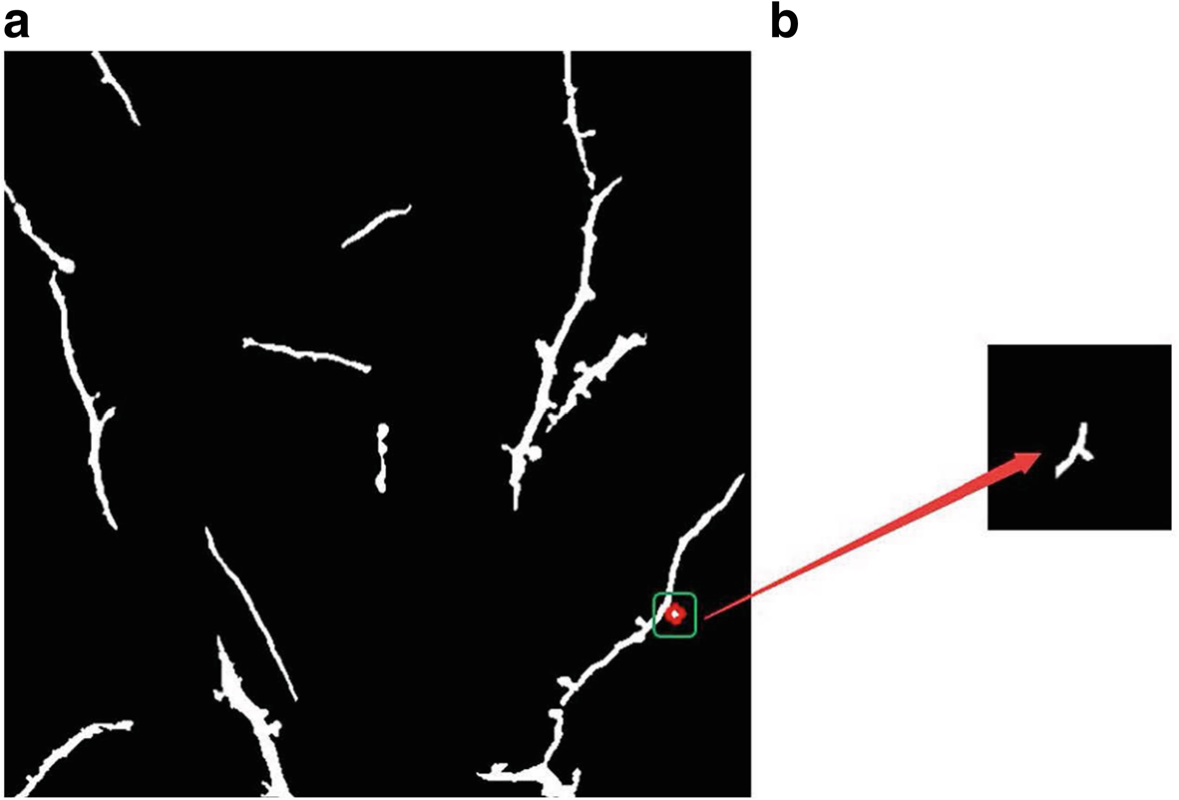
Illustration of filtering spines on points. **a** binary image of dendrites (left) (**b**) structure centered on suspected spine marked by red circle (right)

The transgenic mouse used in this study is YFP-H line, in which a subset of layer 5 cortical neurons express YFP. Therefore, YFP signals in these images contain both dendrites and axons. When searching for dendritic spines, it is essential to determine whether these points are on an axon. For each of the structures that centered on suspected spines with a proper size, illustrated in Fig. 11, we take the ratio of its area to its perimeter and average intensity as judging criteria.

**Fig. 12:**
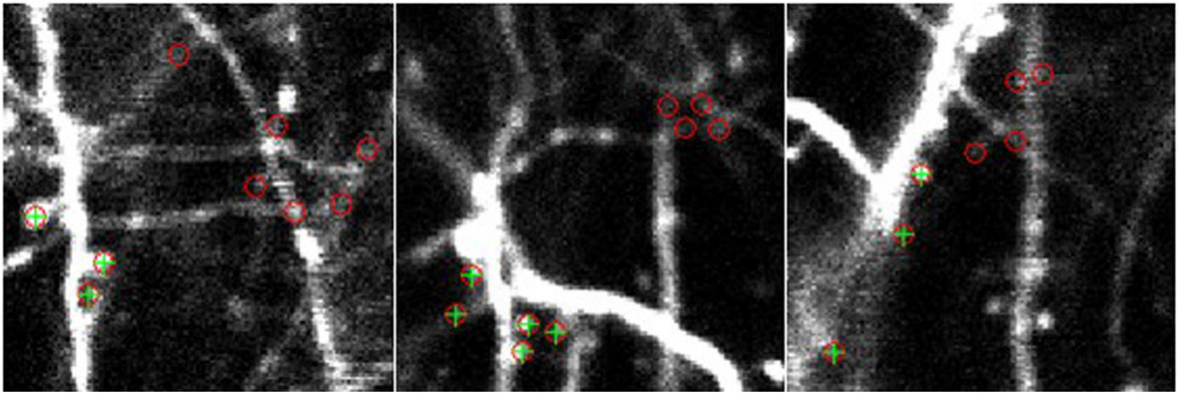
Comparison of results before and after screening on three different layers



As shown in Fig. 12, the positions marked with red circles are the results before screening and the positions marked with green plus sign are results after screening. The positions only marked with red circle are likely locations of axons, rather than spines. The detail is presented in Algorithm 7.

### Detection of synapses

Through the discussion in the previous two sections, we obtained the position of the axonal boutons and dendritic spine in the two-photon image stacks. As mentioned in above section, the presynaptic part is located on an axon and the postsynaptic part is located on a dendrite in mostly synapses. Then, it is reasonable to get the locations of the synapses on 2D by integrating the locations of the axon boutons and dendritic spines. Specifically, we calculate the distance between the axon and dendritic spine to determine whether the two are overlapping. Furthermore, we can count the synapses on 3D based on the detection in continuous 2D images. As shown in Algorithm 8, for each synapse in the 2D image, find its nearest synapse in the next layer. If this synapse is also the nearest of the synapse in the next layer, and the distance between them is close enough, these two synapses are the same synapse in the view of 3D.



## Experimental results

**Fig. 13:**
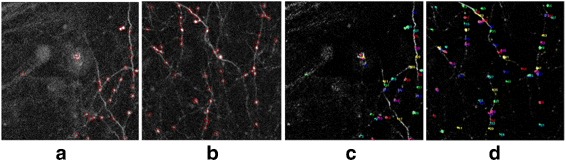
Illustration of true axonal boutons and detected axonal boutons. **a**, **b** True axonal boutons in Layer 1 and Layer 20, respectively. **c**, **d** Detected axonal boutons in Layer 1 and Layer 20, respectively

In order to demonstrate the effectiveness of the proposed algorithm, we show two axon images corresponding to layer 1 and layer 20, with the axonal boutons indicated by red circles marked by experienced neurobiologists in Fig. 13 a and b. The corresponding experimental results detected by our algorithm are shown in Fig. 13 c and d. The ground truth of synapses, axons and dendrites were redundantly marked by three students, and disagreements are decided by another biologist.

The manual annotation process lasts about 2 days. The round-like structures and the structures shown in Fig. 7 were labeled as axonal boutons and dendritic spines respectively, while the spine-bouton pairs were marked as synapses.

We conducted some experiments on other layers and recorded the number of axonal boutons in ground truth. By comparing the detected result with the corresponding ground truth, we respectively determined the number of redundant and missing axonal boutons in different layers as listed in Table 1.

**Table 1:** The numerical analysis of experimental result on detected axonal boutons in each layer

Image	Manual	Our method		
		Total	False positive	False negative
layer 1	29	33	4	0
layer 20	50	54	6	2
layer 40	58	62	5	1
layer 60	90	94	7	3
layer 80	86	89	5	2
layer 100	57	62	5	0
Average (/perception)	61.7	65.8	5.3	1.3

**Fig. 14:**
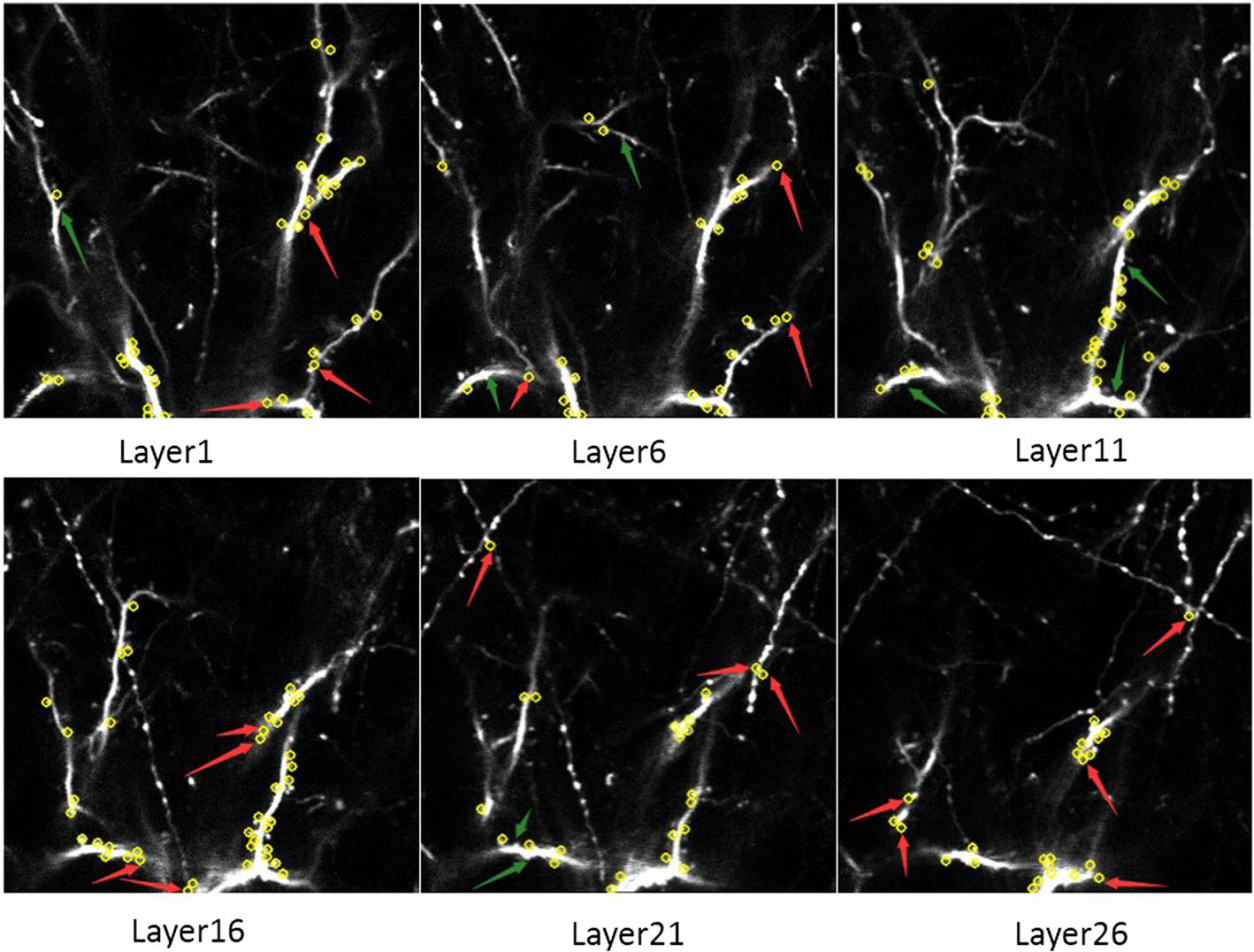
Partial results of dendritic spines detection in Layer 1 to Layer 26. The red arrows point to the location of false positives and the the green arrows point to the location of false negatives

To illustrate the effectiveness of the proposed algorithm, we show partial results of the dendritic spines detection from Layer 1 to Layer 25 in Fig. 14.

As shown in Table 2, we recorded the number of dendritic spines in ground truth and the number of false positive and missing dendritic spines, which were obtained by comparing the detected result with the corresponding ground truth, for several layers.

**Table 2:** Results of detection dendritic spines on several layers

Image	Manual	Our method		
		Total	False positive	False negative
layer 1	31	33	3	1
layer 6	23	24	3	2
layer 11	40	37	0	3
layer 16	35	39	7	0
layer 21	22	23	5	0
layer 26	18	23	5	0
Average (/perception)	34	29.8	3	0.8

**Fig. 15:**
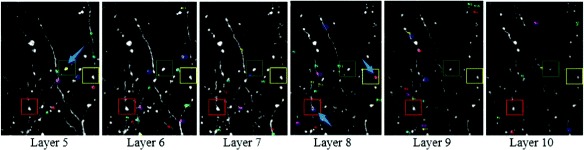
Axonal boutons marked in 3D view. Three different axonal boutons indicated by the red, blue and yellow rectangles with random indexes and colors in layers 5-10 and are solely marked by the blue arrows

**Fig. 16:**
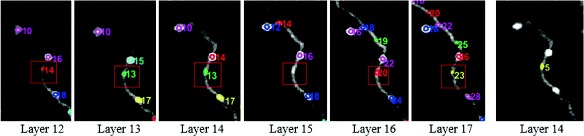
The same axonal bouton appears in the image stack. The axonal boutons in the red rectangles with random indexes and colors are the same bouton that appears in layer 12 to layer 17. It is omitted in layer 15 but is marked solely in layer 14

In Fig. 15, we can see that one axonal bouton indicated by green rectangles arises in layers 5-10 but is only marked in layer 5. Analogously, two axonal boutons are respectively indicated by red rectangles and yellow rectangles are solely marked in layer 8. This method can count the axonal boutons precisely in 3D because it considers the multi-layer information. A specific example in Fig. 16 can account for it.

**Fig. 17:**
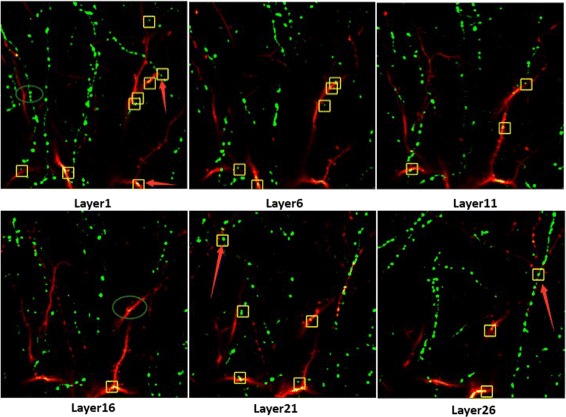
Partial results of synapse detection in Layer 1 to Layer 26. The red arrows point to the location of false positives and green eclipse point to the location of false negatives

The partial results of synapse detection from Layer 1 to Layer 26 are shown in Fig. 17 and the experimental results of synapse detection are shown in Table 3. The green ellipses mark the location of false negatives and the red arrows point to the location of false positives.

**Table 3:** Results of detection synapse on several layers

Image	Manual	Our method		
		Total	False positive	False negative
layer 1	8	2	3	1
layer 6	5	0	3	0
layer 11	2	0	0	0
layer 16	1	0	7	1
layer 21	5	0	5	1
layer 26	3	1	5	0
Average (/perception)	2.7	2.8	0.4	0.3

**Fig. 18:**
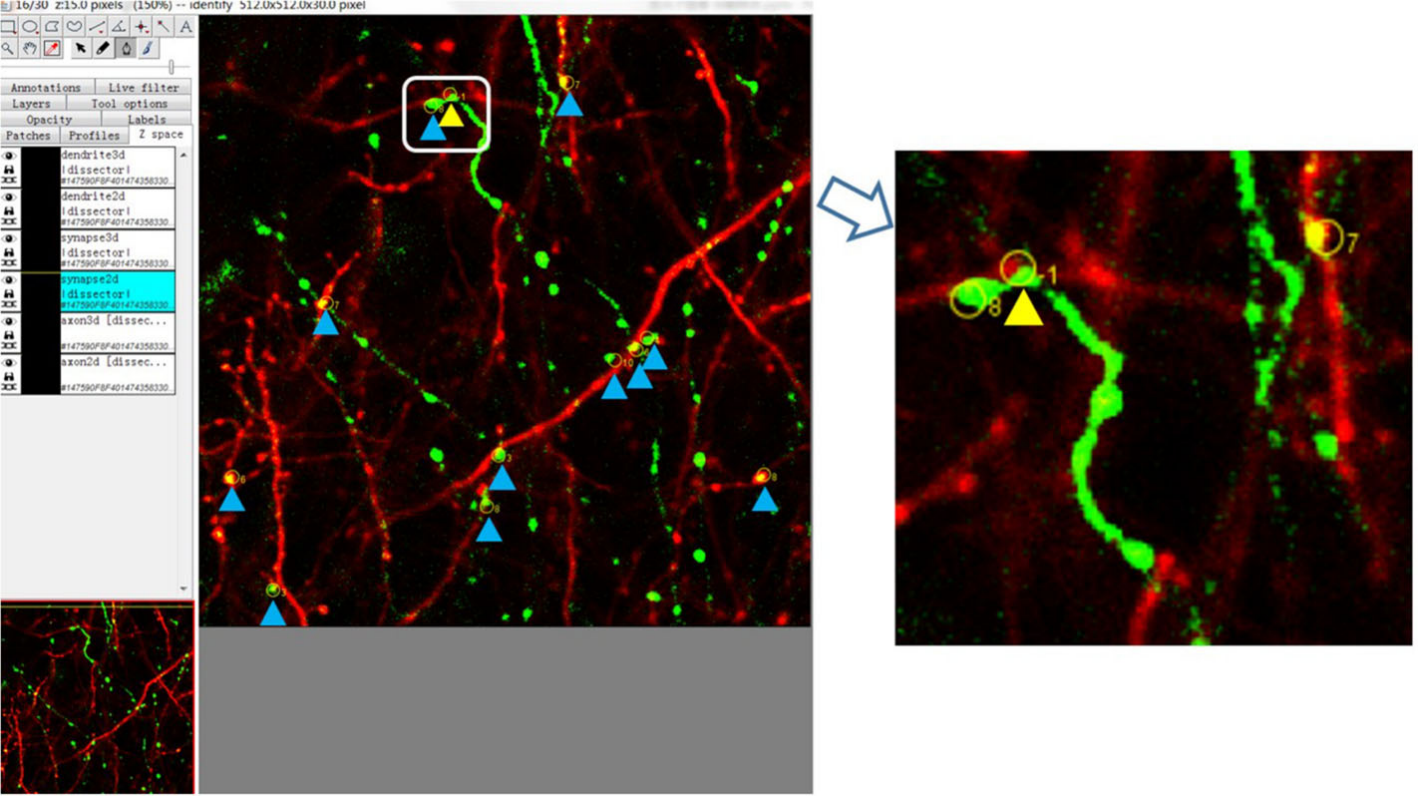
GUI presentation. left: The positions located by the automatic method (marked by blue triangles) and manually marked position (marked by a yellow triangle); right: Corresponding enlarged view of the manually marking position

We have integrated the proposed method of identifying axonal boutons, dendritic spines and synapses with TrakEM, a plugin of ImageJ. This automates synapse analysis process. The left subgraph in Fig. 18 shows the 2D synapse positions, in which synapses are marked by yellow circles. It also provides interactive function, which makes it easy to proofread the detection results. Furthermore, we marked the positions located by the automatic method and by manual annotation with blue triangles and a yellow triangle respectively. The right subgraph of Fig. 18, which was extracted from the left subgraph, and shows the manually marked position (marked by a yellow triangle) with a value of -1.

## Discussion

In vivo two-photon microscopy has been widely used to study structural plasticity of axonal boutons and dendritic spines in live animals. Recently, Yang et al. [19] simultaneously labeled and imaged long-range projecting axons and local dendrites, and studied the turnover dynamics of boutons, spines, and synaptic contacts. This dual-color two-photon imaging method allows in vivo examination of synaptic dynamics in specific neural pathways. However, manual annotation of synaptic contacts is time-consuming and prone to bias. The efficiency of synapse detection will be greatly improved by replacing the manual method with automatic method. The automated method can also be used for bouton and spine detection.

As can be seen from the original image in Fig. 1, the structures of axons and dendrites are not significant enough to cause them to be confused with the ambient noise. Therefore, it is necessary to carry out image enhancement to improve the accuracy of detection.

**Fig. 19:**
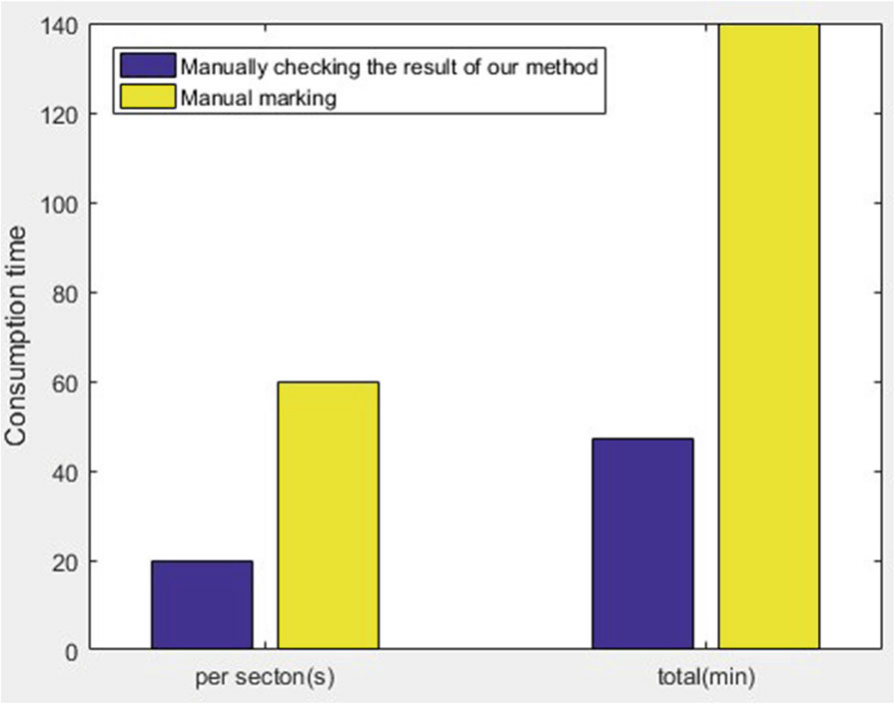
The time spent on manually checking the results of the automatic algorithm and manual annotation

There are 140 two-photon images in total, each of which is 512-by-512 in size with a *x*-*y*-*z* resolution of 137 × 137× 700 nm/pixel. The time spent on manually checking the results of the automatic algorithm and manual annotation are shown in the following bar graph in Fig. 19. We can notice that our approach is much more efficient than manual annotation, especially advantageous if the data volume is larger.

**Fig. 20:**
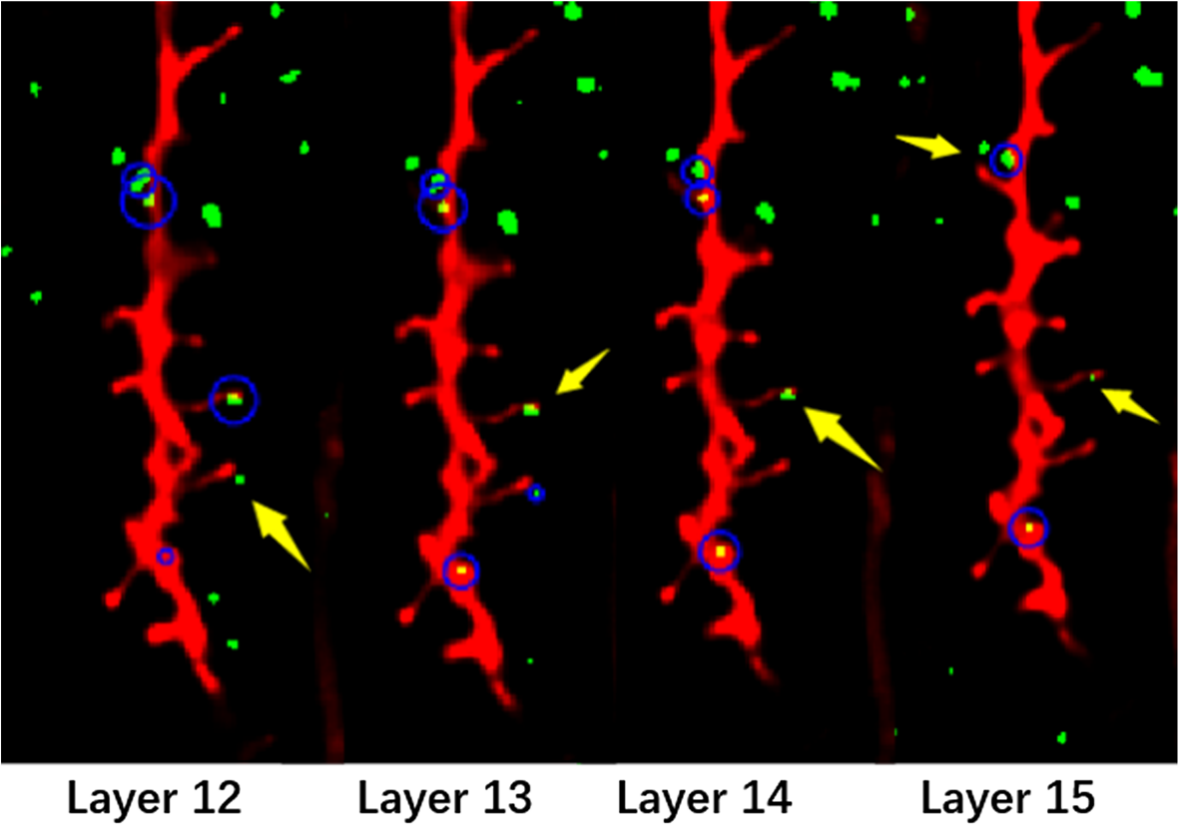
Synapses detection results on Data B

Besides, we have tested our method to another data provided by Beijing Normal University (refered to as Data B) and obtained satisfactory detection results. This dataset provides two-photon image data from neurons in the tbasal ganglia of aeniopygia guttata. The volume of the dataset is 53.3 *μ* *m*×53.3 *μ* *m*×5.6 *μ* *m* and slice thickness is 0.2 *μ* *m*. The size of per image in 2D is 1024 *pixels*×1024 *pixels*. Some of the detection results are shown in Fig. 20, in which the green part are axons and the red part are dendrites. The positions of the candidate synapses detected using our pipeline are denoted by blue circles, while the probable missing synapses are indicated by yellow arrows. We detected all 12 synapses in 3D precisely.

Applying our method to a new dataset requires determining the parameters of image enhancement, ie. the radius of axonal bouton and the radius of lines structure of dendrites.

In [29], Yi Zuo et al. found that, using in vivo two-photon imaging, experienced-dependent elimination of postsynaptic dendritic spines in the cortex was accelerated in ephrin-A2 knockout mice and ephrin-A2 regulates experience-dependent, N-methyl-Daspartate (NMDA) receptor-mediated synaptic pruning through glial glutamate transport during maturation of the mouse cortex. In [30], Ajmal Zemmar et al. tested the effects of Nogo-A neutralization on synaptic plasticity in the motor cortex and on motor learning in the uninjured mature Central nervous system (CNS). According to a series of statistics, such as numbers of dendrites, spines and axons, they concluded that anti-Nogo-A-mediated enhancement of structural and functional synaptic plasticity enlarges the memory capacity per synapse, leading to improved motor learning in vivo. Data analysis in these studies can benefit from our proposed method. Our approach will greatly facilitate data analysis related to dendrite, axon and synapse imaging.

## Conclusion

We presented a novel strategy for identifying axon boutons, dendritic spines, and synapses in in vivo two-photon images. For continuous sequence image stack, we can also count the amount of them in 3D by analyzing the context cues of the detected synapses. This approach will help neuroscientists automatically analyze and quantify the formation, elimination and destabilization of the axonal boutons, dendritic spines and synapses. But it is not yet possible to extract the morphology of synapses. One of our future directions is to get synaptic shapes in 3D.

### Appendix 3D deconvolution

The 3D deconvolution operation implemented in ImageJ [22] consists of the following steps:

1. Download the software ImageJ. Then download the following files: Diffraction_PSF_3D.class, Diffraction_PSF_3D.java, Iterative_Deconvolve_3D.class, and Iterative_Deconvolve_3D.jave. Next, put files in the plugins folder;
2. Run ImageJ and load the original axon image stacks;
3. Open the Diffraction PSF 3D plugin. Fill the form with the related parameters and compute the point-spread function (PSF);
4. Open the Iterative Deconvolve 3D plugin. Select the generated PSF and original axon image stacks, then input the iteration times and generate the deconvolved axon image stacks.

### Appendix A

Model the intensity of axonal bouton using a three-dimensional Gaussian surface:

$$R(x,y) = C\exp\left(- \frac{{{{\left(x - {x_{0}}\right)}^{2}} + {{\left(y - {y_{0}}\right)}^{2}}}}{{2{\delta^{2}}}}\right). $$

The partial derivatives *R*_*xx*_,*R*_*xy*_,*R*_*yx*_,*R*_*yy*_ can be computed as follows:

6 $$  \begin{array}{l} {R_{xx}}(x,y) = R(x,y)\left(\frac{{{{\left(x - {x_{0}}\right)}^{2}}}}{{{\delta^{4}}}} - \frac{1}{{{\delta^{2}}}}\right)\\ {R_{xy}}(x,y) = {R_{yx}}(x,y) = R(x,y)\frac{{\left(x - {x_{0}}\right)\left(y - {y_{0}}\right)}}{{{\delta^{4}}}}\\ {R_{yy}}(x,y) = R(x,y)\left(\frac{{{{\left(y - {y_{0}}\right)}^{2}}}}{{{\delta^{4}}}} - \frac{1}{{{\delta^{2}}}}\right). \end{array}  $$

Then the eigenvalues *λ*_*a*_(*x,y*) of the Hessian matrix are solved as follows:

7 $$\begin{array}{@{}rcl@{}}  \begin{array}{llll} \lambda_{a}(x,y) &=&R(x,y)\left(\!\!\vphantom{\sqrt{{{\left({{\left(x-{x_{0}}\right)}^{2}}+{{\left(y-{y_{0}}\right)}^{2}}+2{\delta^{2}}\right)}^{2}}-4{\delta^{4}}}}\left({(x-x)^{2}}+{\left(y-{y_{0}}\right)^{2}}-2{\delta^{2}}\right)\right.\\ &&\left.\pm \sqrt{{{\left({{\left(x-{x_{0}}\right)}^{2}}+{{\left(y-{y_{0}}\right)}^{2}}+2{\delta^{2}}\right)}^{2}}-4{\delta^{4}}}\right)/2{\delta^{4}}, \end{array} \end{array} $$

### Appendix B

For clarity of presentation, we choose the cross section of *y*=*y*_0_. The Gaussian curve corresponding to the pixel value of cross section *y*=*y*_0_ is

8 $$ R(x) = C\exp\left(- \frac{{{{\left(x - {x_{0}}\right)}^{2}}}}{{2{\delta^{2}}}}\right).  $$

And the edge point (*x* ^∗^,*y*_0_) satisfies

9 $$ \left(x^{\ast}-x_{0}\right)^{2}+\left(y_{0}-y_{0}\right)^{2}=r^{2}  $$

Additionally by the definition in [23], the edge point (*x* ^∗^,*y*_0_) in (8) also satisfies the equation *R* ^′′′^(*x* ^∗^,*y*_0_)=0. After some lengthy calculations, we have

10 $$ R\left(x^{\ast}\right)\left({\left(x^{\ast} - {x_{0}}\right)^{3}} - 3{\delta^{2}}\left(x^{\ast} - {x_{0}}\right)\right) = 0.  $$

A suitable solution is

11 $$ x^{\ast} = {x_{0}} + \sqrt 3 \delta.  $$

According to Eqs. (9) and (11), we conclude that $\delta =r/\sqrt 3 $ is a good choice to identify the axonal boutons.

### Appendix C

According to [23], the magnitude of the second derivative of the extracted position is always maximum at the line position. Then, for a fixed *y*=*y*_0_, the third derivative of Formula(4)

$$I(x') = {C_{den}}exp\left(- \frac{{x'^{2}}}{{2{\sigma^{2}}}}\right) = {C_{den}}exp\left(- \frac{{{{\left(x\cos \theta - y\sin \theta \right)}^{2}}}}{{2{\sigma^{2}}}}\right) $$

can be written as:

$$ - \frac{{I\left(x_{0}\cos \theta - y_{0}\sin \theta\right)}}{{{\sigma^{4}}}}\left(x_{0}\cos \theta - {y_{0}}\sin \theta \right)\left[{\left(x_{0}\cos \theta - {y_{0}}\sin \theta \right)^{2}} - 3{\sigma^{2}}\right] = 0. $$

Then we can obtain

$$x_{0}\cos \theta - {y_{0}}\sin \theta = \sqrt 3 \sigma. $$

With additional efforts, as illustrated in Fig. 9(a), we can obtain the relationship between the variance *σ* and the radius of the linear structure *w*:

$$\sigma = w/\sqrt 3. $$

### Appendix D

For *I* as shown in Formula(4), the partial derivatives *I*_*xx*_,*I*_*xy*_,*I*_*yx*_, and *I*_*yy*_ can be computed as follows:

12 $$ \begin{array}{l} {I_{xx}}(x,y) = I(x,y)\left[ \frac{{{{\cos }^{2}}\theta }}{{{\sigma^{4}}}}{\left(x\cos \theta - y\sin \theta \right)^{2}} - \frac{{{{\cos }^{2}}\theta }}{{{\sigma^{2}}}}\right]\\ {I_{yy}}(x,y) = I(x,y)\left[ \frac{{{{\sin }^{2}}\theta }}{{{\sigma^{4}}}}{\left(x\cos \theta - y\sin \theta \right)^{2}} - \frac{{{{\sin }^{2}}\theta }}{{{\sigma^{2}}}}\right] \end{array}  $$

13 $$ {I_{xy}}(x,y) = {I_{yx}}(x,y) = I(x,y)\left[ \frac{{\sin \theta \cos \theta }}{{{\sigma^{2}}}}+\frac{{\sin \theta \cos \theta }}{{{\sigma^{4}}}}{\left(x\cos \theta - y\sin \theta \right)^{2}}\right].  $$

Then we can get the eigenvalues of $H(x,y) = \left ({\begin {array}{cc} {{I_{xx}}(x,y)}&{{I_{xy}}(x,y)}\\ {{I_{yx}}(x,y)}&{{I_{yy}}(x,y)} \end {array}} \right)$:

14 $$ \lambda_{d}(x,y) = - \frac{1}{{{\sigma^{2}}}}\exp \left(- \frac{{{{\left(x\cos \theta - y\sin \theta \right)}^{2}}}}{{2{\sigma^{2}}}}\right)  $$

**Fig. 21:**
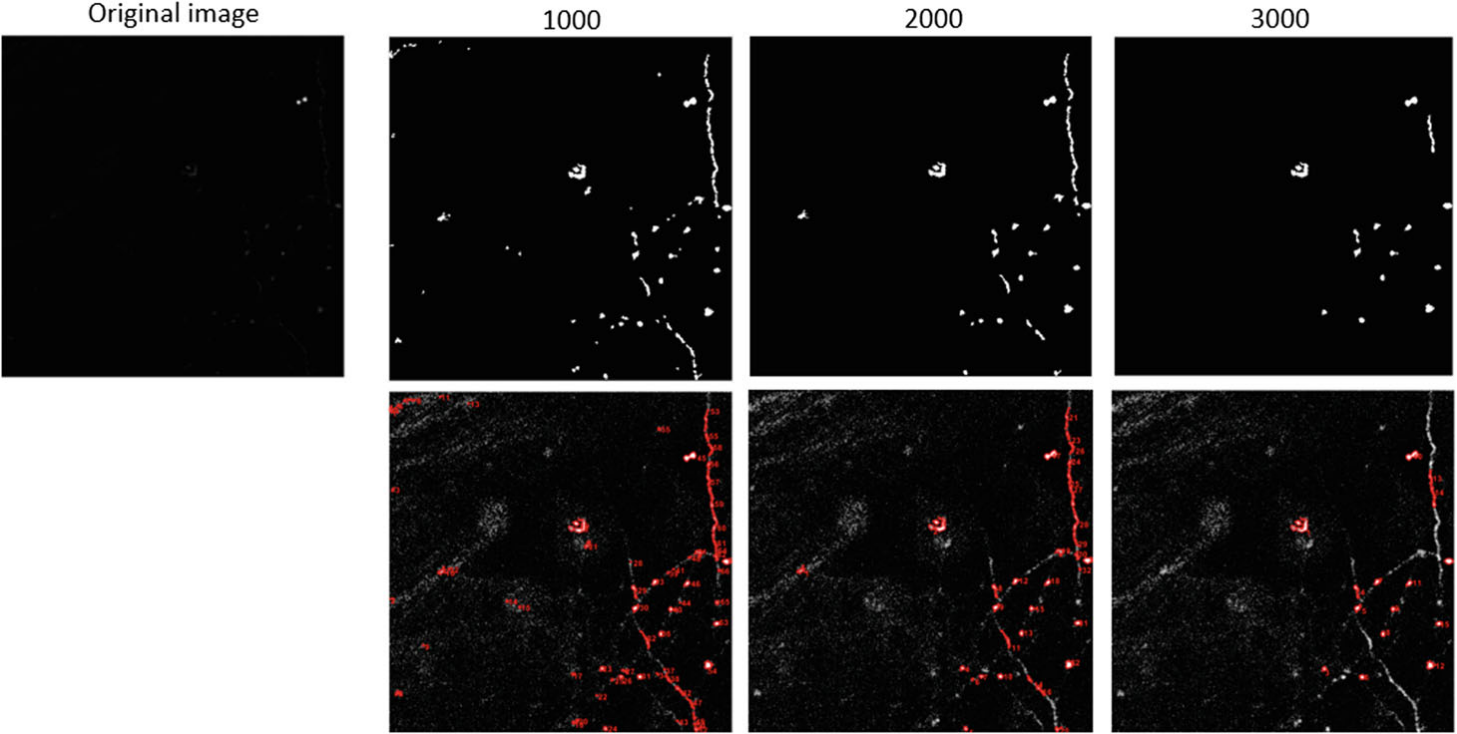
Comparison of segmentation with and without image region enhancement

**Fig. 22:**
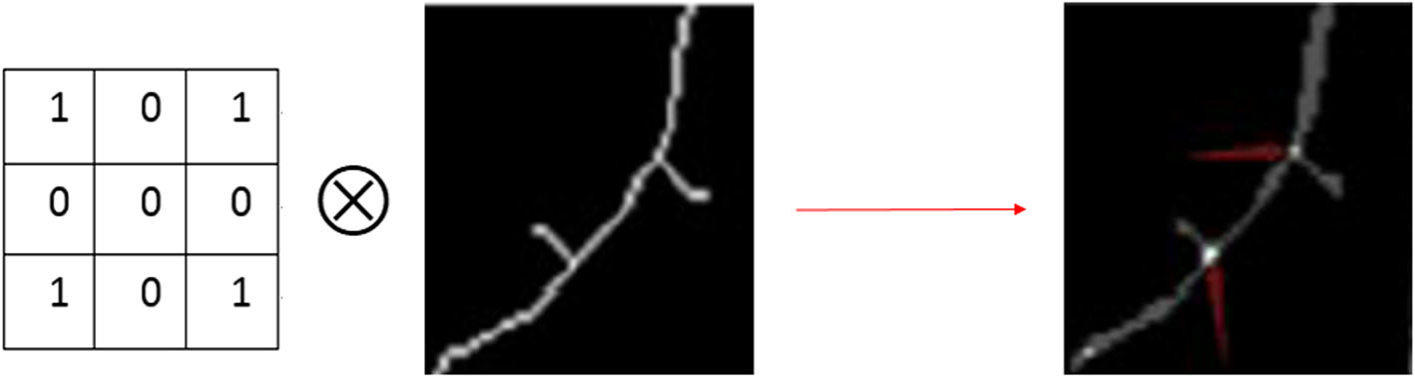
Process of finding branch points. Left: kernel; Middle: skeleton of dendrite; Right: Convolution result

### Appendix E. *Comparison of segmentation with and without image region enhancement*

To justify the use of the image region enhancement on boutons, some useful experiments are conducted. We use three different thresholds, 1000, 2000, 3000, for direct segmentation. Above figures provide direct segmentation results without image region enhancement on boutons. Below figures reserve the final segmentation regions containing local maximum value. From these figures, we conclude that a small threshold will reserve the bright axon shaft, whereas a big threshold will eliminate the weak axonal boutons. For these reasons, we propose to use the image region enhancement method to extract the disk-like structure. The original experiment results demonstrated the effective of our proposed method.

### Appendix F. *Process of finding branch points*

We show the process of finding branch points in the following figure. The kernel is a 3-by-3 filter with an intensity value of 0 for 4 vertices and 1 for the rest positions. And the points on the convolution result, with an intensity value equal or greater than 4 are considered as branch points.

## Abbreviations

CNS: Central nervous system
DAMAS: Deconvolution approach for the mapping of acoustic sources
GFP: Green fluorescent protein
NMDA: N-methyl-Daspartate
PSF: Point-spread function
YFP: Yellow fluorescent protein
